# Research Progress of the Antiviral Bioactivities of Natural Flavonoids

**DOI:** 10.1007/s13659-020-00257-x

**Published:** 2020-09-18

**Authors:** Lin Wang, Junke Song, Ailin Liu, Bin Xiao, Sha Li, Zhang Wen, Yang Lu, Guanhua Du

**Affiliations:** 1 Beijing Key Laboratory of Drug Targets Identification and Drug Screening, Institute of Materia Medica, Chinese Academy of Medical Sciences and Peking Union Medical College, 1 Xian Nong Tan Street, Beijing, 100050 China; 2 Laboratory of Clinical Pharmacy, Ordos Central Hospital, Ordos School of Clinical Medicine, Inner Mongolia Medical University, Ordos, 017000 China

**Keywords:** Antiviral bioactivities, Natural flavonoids, Cellular and molecular mechanisms, Therapeutic applications

## Abstract

Flavonoids are now considered as an indispensable component in a variety of nutraceutical and pharmaceutical applications. Most recent researches have focused on the health aspects of flavonoids for humans. Especially, different flavonoids have been investigated for their potential antiviral activities, and several natural flavonoids exhibited significant antiviral properties both in vitro and in vivo. This review provides a survey of the literature regarding the evidence for antiviral bioactivities of natural flavonoids, highlights the cellular and molecular mechanisms of natural flavonoids on viruses, and presents the details of most reported flavonoids. Meanwhile, future perspectives on therapeutic applications of flavonoids against viral infections were discussed.

## Introduction

Flavonoids comprise one of the largest groups of secondary metabolites found in biologically active plants, including vegetables, fruits, seeds, nuts, wine, and tea. Flavonoids are low molecular weight compounds with a simple 15 carbon backbone, and there are more than 9000 varieties of flavonoids that have been structurally identified. The natural flavonoids are an important source of medicines [1].

**Fig. 1:**
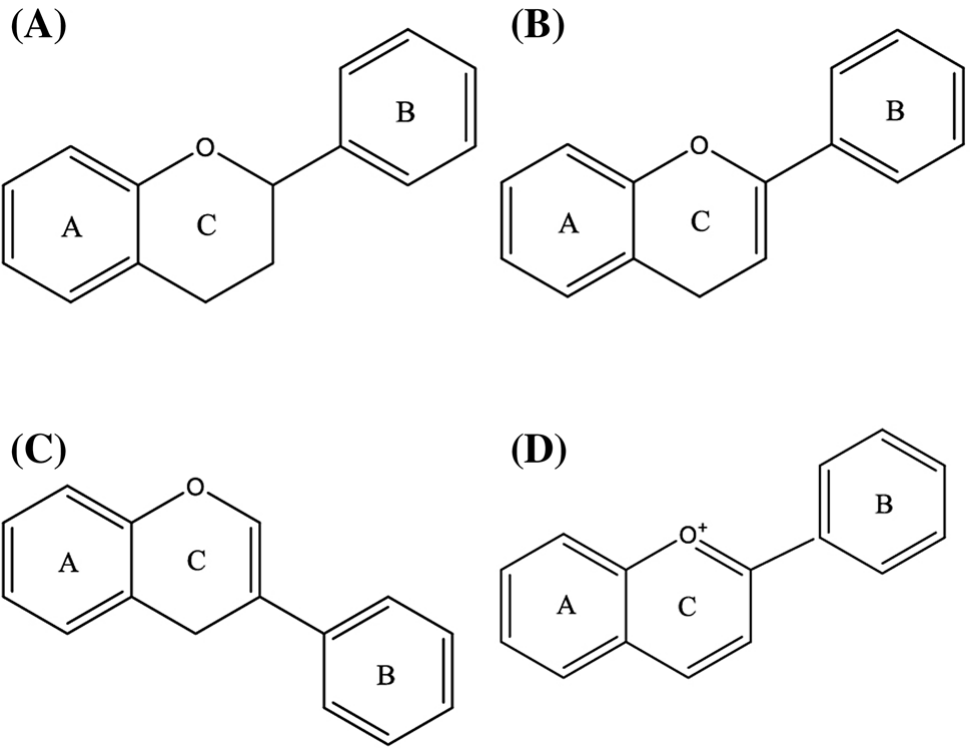
The basic scaffolds of flavonoids. **a** The scaffold of flavanones and catechins; **b** the scaffold of the flavones and flavonols; **c** the scaffold of isoflavone; **d** the scaffold of anthocyanins. The structures of aurones and biflavones were not involved in this figure since their structures containing some special elements which were described in the article

Typically, flavonoids are divided into flavones, flavonols, flavanones, flavanonols, flavanes, flavanols, chalcones, anthocyanidins, aurones, isoflavones, biflavones [2]. The carbon atoms in flavonoid molecules are assembled in two benzene rings, commonly denoted as A and B, which are connected by an oxygen-containing pyrene ring. A common part of the chemical structure of all flavonoids is the carbon skeleton based on the flavan system (C6–C3–C6) (Fig. 1). Aurone is a type of flavonoid with a heterocyclic ring containing a benzofuran element while biflavonoids are dimers of flavonoid moieties linked by a C–C or C–O–C bond. Condensation of A and B ring leads to the formation of chalcone, which undergoes cyclization involving isomerase and forms flavanone, the initial compound for the synthesis of other group flavonoids [3]. Although the various classes of flavonoids possess different structures, all flavonoids appear multi-bioactivities and complex roles in the system of biology.

Most flavonoids, except for the subclass of catechins, are present in plants bound to sugars as β-glycosides. The common sources of natural flavones were the vegetables such as Chamomile tea (*Matricaria chamomilla*), leaves of parsley (*Petroselinum crispum*), celery (*Apium graveolens*) and spinach (*Spinacia oleracea*), roots of plants, propolis, and honey and so on [4]. Even the flavonoids could obtain from various of food and vegetables, the molecules with different structures are of different bioactivities. Since the first report in 1938, flavonoids were described as a broad spectrum of biological activities such as anti-inflammation, antioxidant, antibacterial, antiviral, anticancer, and neuroprotection [5]. In this review, we made a literature retrieval for the anti-virus activity of flavonoids. The antiviral activity of flavones was studied and reported from the 1990s, when apigenin showed synergistic effects to acyclovir on herpes simplex virus types 1 and 2 (HSV-1 and HSV-2) in cell culture. Recently, flavones are reported the inhibitory activity on viruses, including A/FM1/1/47(H1N1), H3N2, H5N1 (strain A/Thailand/Kan-1/04), HBV, HCV, HIV, dengue virus (DENV-2), Sendai virus, Zika virus, Coxsackie virus (CVB3) and Japanese encephalitis virus (JEV) [6]. Especially, the latest study showed flavones efficiently inhibited SARS-CoV [7]. However, numerous positive findings have been reported on the in vitro efficacy of flavonoids, but less promising results have been obtained for most compounds in in vivo studies due to poor bioavailability of flavonoids. The low solubility of flavonoid aglycones in water, coupled with its short residence time in the intestine as well as its lower absorption, save humans from the acute toxic effects via the consumption of flavonoids, except for a rare occurrence of allergy [8]. Therefore, the efforts in enhancing the bioavailability of flavonoids upon intake by humans are vitally necessary in order to develop these natural compounds into potential antiviral drugs.

Generally speaking, the absorption of the dietary flavonoids liberated from the food will depend on their physicochemical properties such as molecular size, configuration, lipophilicity, solubility, and pKa [9]. In addition, flavonoid protein interactions are involved in flavonoid bioavailability. Depending upon structure, the flavonoid can be absorbed from the small intestine or has to go to the colon, where they are metabolized via microbial catabolism, conjugation in liver and enterocytes. Due to these conjugation reactions, no free flavonoid aglycones can be found in plasma or urine, except for catechins [10]. The sugar moiety of flavonoid glycosides is an important determinant of their bioavailability [11].

## Overview of the Research on the Antiviral Effects of Flavonoids

**Fig. 2:**
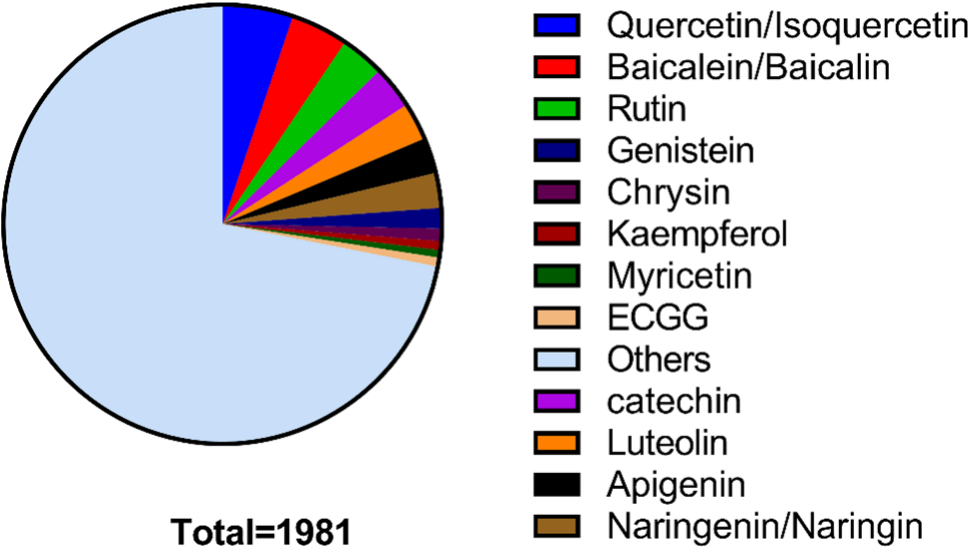
Literature review on the antiviral activities of natural flavonoids

Based on the literature published in the international journals, up to May 2020, more than 1000 researches on the anti-virus activities in vivo and in vitro and 100s of natural flavonoids have been tested in different viruses. But, only about decades were focused, such as coumarin, luteolin, and so on (Fig. 2).

Generally, all the bioactivities found in the flavonoids could be summarized to some main aspects. Flavonoids, including genistein, catechins, and so on, have been shown to reduce the infectivity of a variety of viruses affecting humans and animals, including adenovirus, HSV, HIV, porcine reproductive and respiratory syndrome virus, and rotavirus [12]. Current results about the mechanisms of action underlying their antiviral properties suggest a combination of effects on both the virus and the host cell. Flavonoids have been reported to affect virus adsorption, entry, replication, viral protein translation, the formation of certain virus envelope glycoprotein complexes, and virus release [13–16]. They also affect a variety of host cell signaling processes, including induction of gene transcription factors and secretion of cytokines [17, 18]. Although enormous promising results were from in vitro experiments, a few in vivo results can partly confirm their in vivo efficacy. Flavonoids possess antiviral properties against a wide range of viruses under both in vitro and in vivo conditions (Table 1).

**Table 1:** The different viruses which inhibited by various flavonoids

Viruses	Model	Flavonoids	References
Influenza virus	MDCK cells	Gallocatechin-7-gallate, catechins, apigenin, luteolin, 3-deoxysappanchalcone, scutellarin, galuteolin, vitexin, chrysin, kaempferol, quercetin, myricetin, rhamnocitrin, rutin, daidzein, genistein, sappanchalcone, baicalein, oroxylin A	Liu et al. [19], Yonekawa et al. [20]
HBV	Vero cells	Myricetin rhamnoside, myricetin-3-α-O-ramnosil (1 → 6)-α-galactoside, 5,3′-dihydroxy-3,6,7,8,4′- pentamethoxyflavone, 5-hydroxy-3,6,7,3′,4′- pentamethoxyflavone	Ortega et al. [21]
HCV	Huh-7.5 cells	Epigallocatechin gallate (EGCG), sorbifolin, pedalitin	Mekky et al. [22]
HIV-1	CD4^+^ NKT cells, T cells	Hesperidin, linarin, catechins, genistein, herbacitrin, naringin, formononetin, biochanin A	Nzuza et al. [23]
HIV-2	Vero cells	Genistein, formononetin, biochanin A	Patra [24]
HSV-1	Vero and CV1 cells	Catechins, genistein,gorvanol A, kaempferol, 5,6,7-trimethoxyflavone, 5,3′-dihydroxy-3,6,7,8,4′- pentamethoxyflavone, 5-hydroxy-3,6,7,3′,4′- pentamethoxyflavone, coumestrol, houttuynoid A, chrysin	Li et al. [25]
HSV-2	Vero cells	Genistein, coumestrol, houttuynoid A	Bús et al. [26]
HPV-1	Human condyloma, Vero cells	Catechins, 5,3′-dihydroxy-3,6,7,8,4′- pentamethoxyflavone, 5-hydroxy-3,6,7,3′,4′- pentamethoxyflavone	Patra [24]
DENV-2	C6/36 Aedes albopictus mosquito cell, hepatocytes (Huh-7)	Quercetin, quercitrin, kaempferitrin, chrysin	Patra [24]
Sendai virus (SeV)	Mice model	Baicalein	Dou et al. [27]
Zika virus (ZIKV)	Vero cells	Baicalein, baicalin, pinocembrin, chrysin, myricetin, luteolin, Epigallocatechin gallate, epicatechin gallate, gallocatechin gallate, quercetin-3-β-O-d-glucoside	Oo et al. [28]
CVB3	Vero cells	Mosloflavone, oroxylin A, norwogonin, epigallocatechin-3-gallate	Patra [24]
JEV	A549 cells, BHK21 cells	Epigallocatechin-3-gallate (EGCG), luteolin, kaempferol	Patra [24]
EBV	Ramos cells	Genistein, quercetin, apigenin, luteolin, baicalein	Granato et al. [29]
Poliovirus	Vero cells	5,6,7-Trimethoxyflavone, 3-methylkaempferol, 3(2H)-isoflavene	Ortega et al. [21]
RSV	Vero cells	Genistein, quercetin, baicalein, baicalin, epigallocatechin-3-gallate, proanthocyanidin	Zhang et al. [30]
Coronovirus	Vero cells	Quercetin, Luteolin, quercetin, quercetrin, kaempferol glycosides	Patra [24]
SARS-CoV	3CL protease activity assay	Daidzein, rutin, genistein, icaritin, genistin, ipriflavone, (−) gallocatechin, (±)-epigallocatechin gallate, puerarin, (−)-epicatechin, glabridin, (±)-catechin, baicalein, diosmin, diosmetin, skullcapflavone II, orientin, acacetin, bacicalin, rhoifolin, hispidulin, sinensetin, oroxin B, pectolinarin, cirsiliol, homoplantaginin, amentoflavone, luteolin, herbaacetin, kaempferol, morin, myricetin, fisetin, quercitrin, queretin, helichrysetin, cardamonin, neodesperidin dishydrochalcone, mangiferin, auraptene	Jo et al. [7]
Human CMV	HEL 299 cells	Genistein, 5,6,7-Trimethoxyflavone	Patra [24]
Rotavirus	MA-104 cells, Caco2 cells	Genistein, epigallocatechin Gallate (EGCG), α-glucosyl hesperitin (GH)	Lipson et al. [31]
Adenovirus	Hep2 cells, SW480 cell, BCC-1/KMC cells	Catechins, genistein, quercetin	Patra [24]
SARS-CoV-2	Vero cells	Baicalein, scutellarein, dihydromyricetin, quercetagetin, myricetin	Liu et al. [32]

## Mechanism of Antiviral Flavonoids

**Fig. 3:**
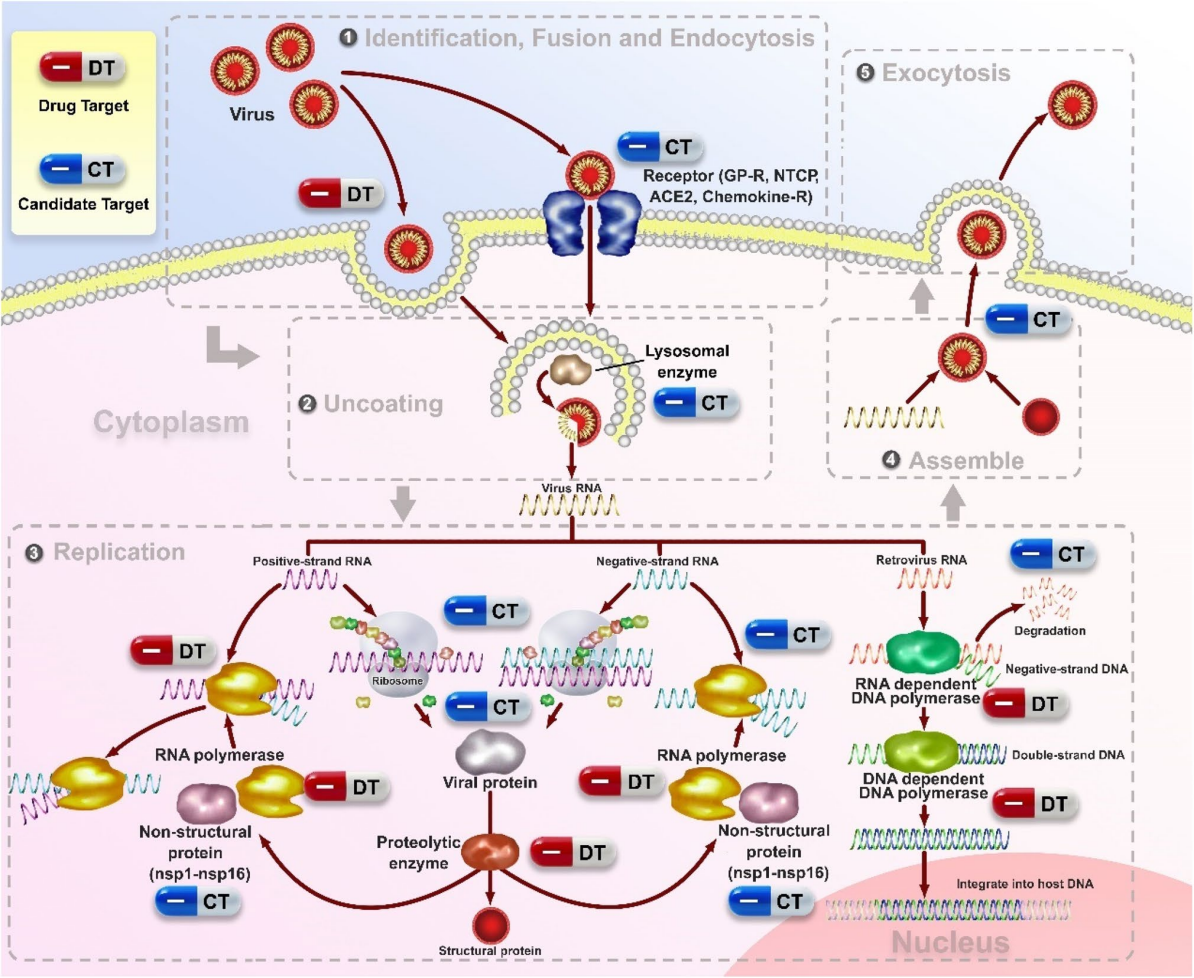
Potential drug targets (DT) and candidate targets (CT) of flavonoids in the viral life cycle. The viral life cycle can be divided into a sequence of stages (attachment and entry; uncoating, replication, assemble, and exocytosis), each of which is a potential site for pharmacologic intervention. Here we showed potential drug targets (DT) and candidate targets (CT) of antiviral flavonoids

In many cases, DNA viruses utilize cellular enzymes for synthesis of their DNA genomes and mRNAs; all viruses utilize normal cellular ribosomes, tRNAs, and translation factors for the synthesis of their proteins. Most viruses commandeer the cellular machinery for macromolecular synthesis during the late phase of infection, directing it to synthesize large amounts of a small number of viral mRNAs and proteins instead of normal cellular macromolecules. The lytic cycle of viral replication includes adsorption, penetration, replication, and release [33] (Fig. 3). The outcome is the production of a new round of viral particles and the death of the cell. According to the lytic cycle of the virus, antiviral drugs can be categorized into the inhibitors of fusion, uncoating, nucleic acid synthesis, integration, protease, and release. Targeting chemokine receptors and glycoprotein (GP)-receptor interactions are also of the most attractive candidates to inhibit viral entry/fusion. Especially, viral enzymes, including RNA polymerase, DNA polymerase, and reverse transcriptase, were considered as alternative targets in many viral infections such as HBV [34, 35]. In addition to previously described viral targets, new classes of antiviral drugs targeting host factors involved in virus replication, virus-cell interactions, and the immune response have been introduced [36].

Many reports on the antiviral activity of naturally occurring flavonoids are available. The structure–function relationship between flavonoids and their enzyme inhibitory activity has been observed. Baicalin was reported to interfere with the viral neuraminidase activity [37]. Flavan-3-o1 was more effective than flavones and flavonones in selective inhibition of HIV-1, HIV-2, and similar immunodeficiency virus infections. Baicalin inhibits HIV-1 infection and replication. Flavonoids such as demethylated gardenin A and robinetin are known to inhibit HIV-1 proteinase. It has also been reported that the flavonoids chrysin, acacetin, and apigenin prevent HIV-1 activation via a novel mechanism that probably involves inhibition of viral transcription [38, 39]. Various combinations of flavones and flavonols have been shown to exhibit antiviral synergism [21]. Kaempferol and luteolin show synergistic effects against herpes simplex virus (HSV). Synergism has also been reported between flavonoids and other antiviral agents. Quercetin is reported to potentiate the effects of 5-ethyl-2-dioxyuridine and acyclovir against HSV and pseudorabies infection [40]. Many flavonoids, namely, dihydroquercetin, dihydrofisetin, leucocyanidin, pelargonidin chloride, and catechin, show activity against several types of viruses, including HSV, respiratory syncytial virus, poliovirus, and Sindbis virus. Quercetin is reported to potentiate the effects of 5-ethyl-2-dioxyuridine and acyclovir against HSV and pseudorabies infection. Studies have displayed that flavonols are more active than flavones against herpes simplex virus type 1, and the activity order from strong to weak was found to be galangin, kaempferol, and quercetin. Zandi et al. studied the anti-dengue virus properties of quercetin, hesperetin, naringin, and daidzein at different stages of DENV-2 (dengue virus type-2) infection and replication cycle [41]. Quercetin was found to be most effective against DENV-2 in cells. In addition, flavonoids were reported anti-inflammation by diminishing inflammatory response and excessive immune response [42]. Therefore, flavonoids protect host cells from damage induced by a viral infection. For instance, selected flavonoids can reduce complement activation, thereby decreasing the adhesion of inflammatory cells to the endothelium and, in general, resulting in a diminished inflammatory response. Another feature of flavonoids is a reduction in the release of peroxidase. This reduction inhibits the production of reactive oxygen species by neutrophils by interfering with 1-antitrypsin activation and the metabolism of arachidonic acid [41, 43].

The flavonoids with anti-virus activities act on various targets, which are known targets with some anti-virus drugs. And some proteins and enzymes which some flavonoids interacted and include in the virus replications may be a candidate target for new anti-virus drugs. (Fig. 3).

## Important Antiviral Natural Flavonoids

### Quercetin and Isoquercitin

Quercetin is widely distributed in angiosperms such as Threevein Astere, Golden Saxifrage, berchemia lineata, gold, rhododendron dauricum, seguin loquat, purple rhododendron, Rhododendron micranthum, Japanese Ardisia Herb and Apocynum. Isoquercitin (IQ), a naturally occurring glycoside of quercetin also known as hirsutrin, isoquercetrin, quercetin­3­glucoside (Q3G), quercetin­3­O­β­d­glucoside. Naturally occurring quercetin compounds are mainly glycosides such as IQ and are commonly found in plants and the human diet.

The quercetin and IQ differ in their structures, bioavailability, absorption, and biological actions. Quercetin and IQ have various kinds of pharmacological functions and are mainly used for treating clinical bronchitis and phlegmatic inflammation. Currently, quercetin/IQ are reported antiviral activities by many researchers (Table 2).

**Table 2:** Anti-virus activities of quercetin/IQ

Virus	Mechanism of action	Dose/concentration	References
A/Udorn/317/72 (H3N2)	Decreases superoxide and LPO associated viral infection	1 mg/day	Kumar et al. [44]
Dengue virus	Inhibits virus replication	20 mg/mL	Keivan Zandi et al. [43]
Japanese encephalitis virus	Inhibits virus adsorption; Interferes virus replication	IC_50_ (212.1 μg/mL)	Jefree Johari et al. [45]
Rhinovirus	Inhibits RV endocytosis and replication and the expression of chemokines and cytokines	10 μM in vitro; 0.2 mg/kg in vivo	Shyamala Ganesan et al. [46]
Mayaro virus	Inhibits virus replication	2 μg/mL	dos Santos et al. [47]
H1N1, H3N2, and H5N1	Binds to Influenza hemagglutinin protein; inhibit viral-cell fusion	IC_50_ (7.756, 6.225, 2.738 μg/mL, respectively)	Wu et al. [48]
Epstein-Barr virus	Induces EBV gene transcription; reduces EBV latency; increases EBV progeny production; inhibits EBV infection	62 μM	Lee et al. [49]
hepatitis C virus	Inhibits HCV replication, specific infectivity; affects virion integrity; hampers the localization of HCV core protein to LDs	50 μM	Ángela Rojas et al. [50]
Influenza A H1N1 (A/PR/8/34)	Inhibits neuraminidase	1.563 μg/mL; 240 mg/kg/days	Liu et al. [51]

### Baicalein

*Scutellaria* *baicalensis* Georgi is a medicinal plant with multiple pharmacological activities. *Scutellaria* *baicalensis* is the main component of Chinese patent medicine preparations for clinical use, such as Shuanghuanglian injection and Qingkailing injection. Baicalin and its active metabolite baicalein are the main pharmacologically active compounds in *Scutellaria* *baicalensis*. Modern research shows that baicalin has certain antiviral activity. Its antiviral pharmacological effect is a concrete manifestation of the heat-clearing and detoxifying effect in the classics of traditional Chinese medicine. With the development of research, details of the antiviral activities of baicalein were reported (Table 3).

**Table 3:** Anti-virus activity of baicalin

Virus	Mechanism of action	Dose/concentration	References
A/FM1/1/47 (H1N1)	Interferes with neuraminidase activity	1.2 μg/mL	Xu et al. [52]
Strain A/Thailand/K (H3N2)	Inhibits virus budding and neuraminidases	IC50:49.6 ± 1.07 μg/mL	Gao et al. [3]
an-1/04 (H5N1)	Interferes with H5N1 replication	IC50:18.79 ± 1.17 μM	Sithisarn et al. [15]
SARS-CoV	3CL^pro^	0.39 μM	Liu et al. (2020)
Zika virus	Inhibits virus replication	0.004 µM	Oo et al. [28]
Dengue virus	Inhibits virus replication	IC50:13.5 ± 0.08 μg/mL	Moghaddam et al. [53]
Sendai virus	Interferes with neuraminidase	0.70 μg/mL	Dou et al. [27]
Japanese encephalitis virus	Interactions with the E protein of DENV2	14.28 µg/mL	Johari et al. [45]
CVB3	Inhibits virus replication	IC50:429.00 ± 22.06 μg/mL	Gao et al. [54]
Japanese encephalitis virus	Direct virucidal activity	14.28 µg/mL	Johari et al. [45]
Human HIV-1	Inhibits HIV-1 induced syncytium formation, HIV-1 p24 antigen, and HIV-1 RT production; inhibits Env-protein mediated fusion of HIV	4.3 μM	Fesen et al. [55]
DENV-2	Inhibits virus replication	1.55 μg/mL	Zandi et al. [45]

### Apigenin

Apigenin, a member of the flavone family, is a nontoxic and nonmutagenic dietary flavonoid found in parsley, artichoke, basil, celery, and other plants. Apigenin (4′,5,7-trihydroxyflavone) contains a hydroxyl group in its B-ring, and hydroxyl groups in its C-ring. The apigenin contained plants are used for the treatment of different diseases and infections like diabetes, dysentery, hepatitis, blennorrhagia, cancer arthritis, inflammation, woods, hemorrhoids, and leishmanial ulcers. Especially, apigenin exhibits various antiviral activities against numerous viruses in vitro and in vivo: enterovirus 71 (EV71), hepatitis C virus (HCV), Human Immunodeficiency Virus (HIV), and adenoviruses. Apigenin exerted inhibitory effects on HCV replication by decreasing mature miR122 expression levels. Apigenin also inhibited FMDV (Foot and mouth disease virus) infection by suppressing IRES-driven translational activity inhibited FMDV infection at the post-entry stage. Apigenin inhibits EBV reactivation into the lytic cycle and virion production by EBV-positive NPC cells. The antiviral activity of apigenin is currently reported, as shown in Table 4.

**Table 4:** Anti-virus activity of apigenin

Virus	Mechanism of action	Dose/concentration	References
EBV	Inhibits expression of EBV lytic proteins, Zta, Rta, EAD, and DNase	200 to 295 μM (24 h); 69 to 158 μM (48 h)	An et al. [56]
African swine fever virus	Inhibits ASFV-specific protein synthesis and viral factory formation	IC50:212.1 ± 11.5 μM	Hakobyan et al. [57]
HCV	Inhibits HCV replication by decreasing mature miR122 expression	5 μM	Shibata et al. [58]
SARS-CoV 3CL^pro^	Inhibits SARS-CoV 3CL^pro^	280.8 μM	Ryu et al. [59]
PEDV	Interferes PEDV replication		Choi et al. [60]
FMD virus	Inhibits cytopathogenic effect and FMDV replication		Qian et al. [61]
HIV	Inhibits CYP3A4, slowdown elimination of PIs		Kehinde et al. [62]
Influenza virus		1.43 μg/mL	Liu et al. [51]
Vaccinia virus	Inhibits VV replication		Chang et al. [63]
PV-2		12.2–13.3 μM	Visintini Jaime et al. [64]

### Luteolin

Luteolin (3,4,5,7-tetrahydroxyflavone) is a pure yellow crystal representing the category of bioflavonoid. It is abundant in various medicinal herbs, fruits, and vegetables, e.g., broccoli, onion, parsley, green peppers, citrus, celery, and chamomile. Luteolin has many beneficial properties, including antioxidant, anti-inflammatory, anticancer, anti-diabetic, and cardio-protective effects and widely used in the development of different traditional medicines for the treatment of diseases. It is also well known to have good effects on anti-angiogenesis, anti-metastasis, anti-inflammation, and estrogenic regulation and can regulate many signal pathways. Besides, luteolin is considered to have potential clinical value for cancer prevention and therapies. Luteolin can obstruct the later stages of the DENV viral life cycle in infected cells by inhibiting the host proprotein convertase furin. Luteolin also exhibits inhibitory effects on Epstein-Barr Virus, Japanese encephalitis virus, HIV-1, Hepatitis B virus, Hepatitis C virus, enterovirus 71, coxsackievirus A16, and chikungunya virus (Table 5).

**Table 5:** Anti-virus activity of luteolin

Virus	Mechanism of action	Dose/concentration	References
DENV	Inhibits proprotein convertase furin	10 mM 100 mg/kg	Peng et al. [65]
EBV	Inhibits viral lytic proteins expression and interferes with Sp1 binding to the IE gene promoters	NA cells (IC50 = 8.6–18.1 μM); HA cells (IC50 = 6–12.3 μM); B cells (IC50 = 6-8 μM)	Wu et al. [66]
Japanese Encephalitis Virus (JEV)	Inhibits JEV replication	IC50 = 4.56 μg/mL	Fan et al. [67]
Influenza virus A/Jinan/15/90 (H3N2)	Inhibits neuraminidase (NA) activities	IC50 = 7.15 μM	Liu et al. [51]
Influenza virus A/Jiangxi/312/2006 (H3N2) A/Fort Monmouth/1/1947 (H1N1)	Interferes with the virus at the early stages of its lifecycle and blocks influenza virus absorption and internalization	IC50 = 6.89 μM	Yan et al. [68]
HBV	Inhibits HBV transcription through ERK-mediated downregulation of HNF4α expression	10–40 μM; 2 mg/kg	Bai et al. [69]
HIV-1	Inhibits HIV-1 activity; infection by abrogating Tat-mediated LTR activity	5–10 μM	Mehla et al. [70]
SARS-CoV	Binds to the surface spike protein of SARS-CoV and inhibits entry of the virus into host cells	EC50 = 10.6 μM	Yi et al. [71]
COVID-19	Inhibits COVID-19 main protease Mpro		Khaerunnisa et al. [72]

### Isorhamnetin

Isorhamnetin (Iso) is a flavonoid compound extracted from the Chinese herb *Hippophae* *rhamnoides* L. Previous studies have revealed its anticancer, anti-inflammatory, and antioxidant activities. What's notable here is the antiviral activity of isorhamnetin. Oral administration of isorhamnetin in mice infected with the influenza A virus significantly decreased lung virus titer by 2-folds and decreased the virus titer in vivo using embryonated chicken eggs. Structure–activity relationship (SAR) showed the methyl group located on the B ring of isorhamnetin might contribute to its strong antiviral potency against the influenza virus in comparison with other flavonoids. In addition, isorhamnetin treatment reduced virus-induced ROS generation and blocked cytoplasmic lysosome acidification and the lipidation of microtubule associated protein1 light chain 3-B (LC3B). The evidence for the anti-virus activity of isorhamnetin was shown in Table 6.

**Table 6:** Anti-virus activity of isorhamnetin

Virus	Mechanism of action	Dose/concentration	In vitro*/*in vivo	References
EV71 virus	Inhibits EV71 RNA replication and protein synthesis	10 mg/kg	In vivo	Dai et al. [73]
H1N1 virus	Reduces virus-induced active oxygen production, blocking cytoplasmic lysosomal acidification and lipid formation of microtubule-associated proteins	1 mg/kg	In vivo	Enkhtaivan et al. [74]
HHV1 virus HHV2 virus	Adheres to the cell surface and reduces the interaction between cells and viruses	100 g/mL	In vitro	Sochocka et al. [75]
Zika virus	Inhibits NS3–NS2B protease	600 µM	*Molecular* *docking* *study*	Sonam et al. [76]

### Isoflavone

Isoflavones are polyphenolic secondary plant metabolites that are produced primarily from members of the Leguminosae. There are hundreds of naturally occurring isoflavones isolated and identified. Common isoflavones include daidzin, genistin, biochanin A, and formononetin. All isoflavones share the 3-phenylchromen-4-one backbone, which is always modified, mainly by O-substituents, glycosides, and/or prenylated derivatives. In plants consumed as part of the human diet (including dietary supplements), the highest concentrations of isoflavones have been observed in soy (*Glycine* *max* L.), red clover (*Trifolium* *pratense*), and kudzu. Isoflavones exhibit antioxidant, anticancer, antimicrobial, anti-inflammatory, antiosteoporotic, and estrogenic properties. Especially, isoflavones and their related flavonoid compounds exert antiviral properties both in vitro and in vivo against a wide range of viruses. Targets of Isoflavones reported affecting virus binding, entry, replication, viral protein translation, and formation of certain virus envelope glycoprotein complexes. Isoflavones also affect a variety of host cell signaling processes, including induction of gene transcription factors and secretion of cytokines (Table 7).

**Table 7:** Anti-virus activity of isoflavones

Isoflavone	Virus	Mechanism of action	Dose/concentration	References
Genistein	Avian leucosis virus	Inhibits the late phase of ALV-J replicative cycle	12.5–100 μM	Qian et al. [77]
	Porcine reproductive and respiratory syndrome virus (PRRSV)	Activation of adaptive immune system pathways		Smith et al. [78]
Genistein	African swine fever virus	Disrupts viral DNA replication, blocking the transcription of late viral genes as well as the synthesis of late viral proteins, reducing viral progeny	IC_50_ = 13 μM,	Arabyan et al. [79]
Genistein	rotavirus	Inhibits rotavirus replication and upregulates AQP4 expression	80 μM	Huang et al. [80]
Genistein	Herpes simplex virus	Inhibits virus replication	40 μM	Argenta et al. [81]
KIN 101	hepatitis C virus (HCV) and influenza virus	Activates the ISG54 promoter mediated nuclear translocation of IRF-3	IC_50_ = 0.2 μM	Bedard et al. [82]
Deguelin	Human cytomegalovirus (HCMV)	Suppresses the production of the infectious virus; inhibits the lytic cycle	250 nM	Nukui et al. [83]
	HIV-1	Inhibits HIV-1 entry into cell lines, primary human CD4^+^ T lymphocytes, and macrophages	IC_50_ = 81.6 ± 4.3 μM	Mediouni et al. [84]
Daidzein	Dengue virus type-2	Inhibits virus replication	IC_50_ = 142.6 μg/mL	Zandi et al. [43]

### Catechin/EGCG (Epigallocatechin-3-gallate)

Catechins are important ingredients from tea leaves and account for more than 75% of the polyphenol compounds in tea leaves. Catechins are members of the group of polyphenol compounds found in many medicinal plants, with a ring and the basic structure of flavan-3-ol and have intensive anti-oxidant and representative physiological activities.The major sources of catechins are *Camellia* *sinensis* (*C.* *sinensis*) and *C.* *assumica*. There are eight catechin: C ((−)-catechin), EC ((−)-epicatechin), ECG ((−)-epicatechingallate), EGC ((−)-epigallocatechin), EGCG ((−)-epigallocatechin gallate), GC ((−)-gallocatechin), CG ((−)-catechingallate), and GCG ((−)-gallocatechingallate). Because of the hydroxyl in the gallate group, Epigallocatechin-3-*O*-gallate (EGCG) and ECG are highly effective free-radical scavengers compared with many other standard anti-oxidants. According to the relationships between structure and antiviral activity of catechin derivatives, the 3-galloyl and 5′-OH group of catechin derivatives appear critical to antiviral activities. Most Catechin/EGCG were reported not to affect cell viability and proliferation but interfered with herpes simplex virus cell penetration and adhesion. Among these catechins, EGCG is the major catechin component of green tea (*Cameria* *sinensis*) and known to possess antiviral activities against a wide range of DNA viruses and RNA viruses. EGCG has been reported to possess a broad spectrum of antiviral activities against DNA viruses such as herpes simplex virus (HSV; Herpesviridae) adenovirus (Adenoviridae), human papilloma virus (HPV; Papovaviridae), and hepatitis B virus (HBV; Hepadnaviridae), and against (+)-RNA viruses such as hepatitis C virus (HCV; Flaviviridae), Zika virus (ZIKV; Flaviviridae), dengue virus (DENV; Flaviviridae), West Nile viruses (WNV; Flaviviridae), Chikungunya virus (CHIKV; Togaviridae), and Porcine Reproductive and Respiratory virus (PRRS; Atteriviridae), and (−)RNA viruses such as human immunodeficiency virus (HIV; Retroviridae), Ebola virus (EBOV; Filoviridae) and influenza virus (Table 8).

**Table 8:** Anti-virus activity of catechin/EGCG

Catechin	Virus	Mechanism of action	Dose/concentration	References
EGCG	HBV	Detrimental to HBV replication by altering lysosomal acidification	25–50 μM	Zhong et al. [85]
EC, ECG, EGC and EGCG	HSV-1/HSV-2	Destructive HSV-1 virions;competitively interacted with virion surface proteins	HSV1:IC99 (IC_50_: 18.3–72.3 μM) HSV2:IC_99_ (IC_50_: 12.5–25 μM)	Isaacs et al. [86] Colpitts et al. [87]
EGCG	EBV	Suppressed the synthesis of lytic protein; inhibited the lytic infection; reducing the DNA binding potency of nuclear antigen; inhibition of the MEK/ERK1/2 and PI3-K/Akt signaling pathways	IC_50_: 250 μM	Weber et al. [88] Liu et al. [89]
EGCG	Adenovirus	Inhibited the attachment of adenovirus by interacting with virion surface proteins	IC_50_:20 µM	Liu et al. [90]
EGCG, EC ECG	HIV1/HIV2	Inhibitory action against HIVRT; competitive inhibitors of the template-primer; noncompetitive inhibitors of Dttp; inhibits HIV entry	EGCG:300 mg/kg/day; IC50:3.44 ± 1.07 µM GCG:2.45 ± 0.36 µM	Yamaguchi et al. [91] Hartjen et al. [92] Rrapo et al. [93]
EGCG	HCV	Inhibitor of the HCV entry and viral RNA replication	IC_50_: 17.9 μM	Chen et al. [94]
EGCG, EC ECG	Influenza A/B	Inhibitory effects on the acidification of endosomes and lysosomes;	EC: > 145.09 μg/mL EGC: 30.49 μg/mL EGCG: 56.49 μg/mL	Imanishi [95] Yang et al [96]
EGCG	DENV, JEV and TBEV	Associated with the DENV2 E protein; destruction of the structure of ZIKV virions	> 100 μM	Carneiro et al. [97]
EGCG	Human T-cell Lymphotropic Virus-1	Reduce the invasive potential of HTLV-1-positive leukemia cells; suppressing Tax expression; inhibiting the activation of NF-kB pathway and induction of MMP-9 transcription in HTLV-1 positive cells	25 μM in HuT-102	Harakeh et al. [98]
EGCG GCC	Rotaviruses enteroviruses	Interfering with virus adsorption; reduced reactive oxygen species (ROS) generation	GCG: 10 μM EGCG:10 μM	Ho et al. [99]
EGCG	Ebola virus (EBOV)	Reduced the production of new viruses via inhibiting HSPS5	10–100 μM	Reid et al. [100]

## Conclusion

The study of flavonoids is complex because of the heterogeneity of the different molecular structures and the scarcity of data on bioavailability. An important effect of flavonoids is the scavenging of oxygen-derived free radicals. Flavonoids possess anti-inflammatory, antiallergic, antiviral, and anticarcinogenic properties from the evidence obtained in vitro experimental systems. However, the clinical application of flavonoids is restricted by its low solubility and poor bioavailability. Although there are lots of advances in the antiviral pharmacology of natural flavonoids, further study is needed to elucidate the effects of flavonoids within the body and the degree and rate of absorption for the evaluation of druggability.

With the development of preparation techniques, newly developed flavonoids preparations exhibit better absorption and thus have higher bioavailability. Since flavonoids are frequently prescribed with other medications, understanding the compatibility of co-administrated drugs is of importance for clinical applications, and requires further research for better clarity. Furthermore, pharmacokinetic changes of flavonoids under different pathological conditions indicate clinical considerations of drug safety and the possible requirement of individualized antiviral therapy.
